# Supplementary material for: Improving magnetic resonance imaging with smart and thin metasurfaces
Source: Sci Rep. 2021 Aug 10;11:16179. doi: 10.1038/s41598-021-95420-w (PMC8355254; doi:10.1038/s41598-021-95420-w)
Supplement: Supplementary file 1 — Supplementary material 1 (pdf 6642 KB) [file 41598_2021_95420_MOESM1_ESM.pdf]

Improving Magnetic Resonance Imaging with Smart and Thin Metasurfaces

# - Supplementary Material -

Endri Stoja[1], Simon Konstandin[2], Dennis Philipp*[2], Robin N. Wilke[2], Diego Betancourt[1], Thomas Bertuch[1], Jürgen Jenne[2,3], Reiner Umathum[2,3], and Matthias Günther[2,4]

[1]Fraunhofer FHR, Fraunhoferstraße 20, 53343 Wachtberg, Germany
[2]Fraunhofer MEVIS, Am Fallturm 1, 28359 Bremen, Germany
[3]Division of Medical Physics in Radiology, German Cancer Research Center DKFZ, Im Neuenheimer Feld 280, 69120 Heidelberg, Germany
[4]MR-Imaging and Spectroscopy, Faculty 01, University of Bremen, Otto-Hahn-Allee 1, 28359 Bremen, Germany
*dennis.philipp@mevis.fraunhofer.de

This file contains supplementary material in the form of extended descriptions, figures, and measurement data.

On theoretical modeling

An approximate theoretical description of the smart metasurfaces can be obtained using the coupled mode theory. For the linear part of the system, the inner metasurface structure, the mode amplitude y_1 depends on an excitation x according to

$$\dot{y}_1 = a_1(\omega_1)\,y_1 + b_1\,x\,, \quad a_1(\omega_1) = i\omega_1 - 1/\tau_r - 1/\tau_i\,, \quad b_1 = \sqrt{2/\tau_r}\,. \tag{1}$$

Here, ω_1 is the resonance frequency, τ_r is the decay time due to radiation losses, and τ_i is the decay time associated with intrinsic losses. The overdot denotes the time derivative. Note that the inverse of the lifetimes τ_i are to be considered as decay rates. The coupling to the excitation is via the radiative term. A similar equation can be constructed for the tuning resonator,

$$\dot{y}_2 = a_2(\omega_2)\,y_2 + b_2\,x\,, \tag{2}$$

but now the resonance frequency ω_2 depends on the resonator's amplitude as well, $\omega_2 = \omega_2(y_2)$. We may assume a linear relation such that $\omega_2 = \Omega_2 + \kappa|y_2|$ with a constant κ that depends on the properties of the chosen non-linear element and a small amplitude limit Ω_2. Finally, the full smart metasurface is described by the coupled, non-linear system of differential equations in the time domain,

$$\dot{y}_1 = \big(i\omega_1 - 1/\tau_{r,1} - 1/\tau_{i,1}\big)y_1 + \sqrt{2/\tau_{r,1}}\,x + iky_2\,, \tag{3a}$$

$$\dot{y}_2 = \big(i\Omega_2 + i\kappa|y_2| - 1/\tau_{r,2} - 1/\tau_{i,2}\big)y_2 + \sqrt{2/\tau_{r,2}}\,x + iky_1\,, \tag{3b}$$

which includes the coupling strength k. Let us be precise regarding the dimension of involved quantities. Certainly, ω is an inverse time, i.e., it is measured in Hz. All τ_i are times, measured in seconds. For the excitation x we have that the dimension of $|x|^2$ is a power, thus for the mode amplitude the dimension of $|y_i|^2$ is an energy. Hence, the dimension of the quotient of mode amplitude and excitation is $\big[|y_i|^2/|x|^2\big] = \mathrm{s}$. The system of equations can be simplified by assuming that the excitation is expressed as $x = |x|\exp(i\omega t)$. Then, by proceeding to the frequency domain the time derivatives are converted into algebraic expressions and the equations decouple such that

$$\begin{aligned}\Big[\big(i(\omega-\omega_1) + 1/\tau_{r,1} + 1/\tau_{i,1}\big)\big(i(\omega - \Omega_2 - \kappa|y_2|) + 1/\tau_{r,2} + 1/\tau_{i,2}\big) + k^2\Big]\,y_2 \\ = \Big[ik\sqrt{2/\tau_{r,1}} + \big(i(\omega-\omega_1) + 1/\tau_{r,1} + 1/\tau_{i,1}\big)\sqrt{2/\tau_{r,2}}\Big]\,|x|\end{aligned} \tag{4}$$

remains to be solved numerically for y_2, and y_1 is subsequently given by

$$y_1 = \Big(iky_2 + \sqrt{2/\tau_{r,1}}\,|x|\Big)\,\big(i(\omega-\omega_1) + 1/\tau_{r,1} + 1/\tau_{i,1}\big)^{-1}\,. \tag{5}$$

To calculate the reflection of the full system, we use

$$R = \frac{\left|-x + \sqrt{2/\tau_{r,1}}\, y_1 + \sqrt{2/\tau_{r,2}}\, y_2\right|^2}{|x|^2}\,. \tag{6}$$

This equation can be used to model our measurements of the S11 parameter with the untuned sniffer coil. The theoretical description fits the experimental results qualitatively when the eight parameters are chosen appropriately. For small excitation strength, there is only one solution for the mode amplitude y_2, whereas for strong excitations three solution branches appear, of which the middle one is unstable, see Fig. S1. Note that all parameters needed for the model, i.e., resonance frequencies, decay rates, and couplings can be given as multiples of the fundamental resonance ω_1. These eight parameters are the fundamental resonances of the two coupled systems ω_1, ω_2, the coupling strength k between the two, a parameter κ to describe the non-linear behaviour of the tuning resonator, and the time scales $(t_{r,1}, t_{r,2}), (t_{i,1}, t_{i,2})$ related to radiation losses and internal losses, respectively. They have to following values for the model of the reflection R, which is shown in supplementary Fig. S1b.

$$t_{r,1} = 3000/\omega_1\,, \quad t_{r,2} = 100/\omega_1 \tag{7a}$$
$$t_{i,1} = 600/\omega_1\,, \quad t_{i,1} = 100/\omega_1 \tag{7b}$$
$$\omega_1 = 136\,\text{MHz}\,, \quad \Omega_2 = \omega_1 + 2\,\text{MHz} \tag{7c}$$
$$\kappa = -0.16\omega_1\,, \quad k = 0.1\omega_1\,. \tag{7d}$$

To model the magnetic field focussing effect, i.e., the SNR enhancement, the following steps are needed in principle. (i) The current distribution in individual wire-resonator unit cells and the tuning resonator is to be modeled / numerically solved for. (ii) Then, using Bio-Savart's law the induced magnetic field at some distance can be calculated. (iii) The obtained magnetic field is to be superimposed on the excitation field and the enhancement is given by

$$\eta(\vec{x}) := \frac{|B_{\text{ind},z}(\vec{x};\omega) + B_{0,z}(\vec{x})|}{|B_{0,z}(\vec{x})|}\,, \tag{8}$$

in which $B_{\text{ind},z}(\vec{x};\omega)$ is $z-$component of the induced magnetic field, which is extremal at the design resonance frequency. In future work, a detailed numerical and mathematical study of this and other (advanced) metasurfaces will be presented including quantitative matching of theoretically modeled, simulated, and measured system characteristics as well as simulations of the signal focusing in MRI experiments.

Additional Figures and Data

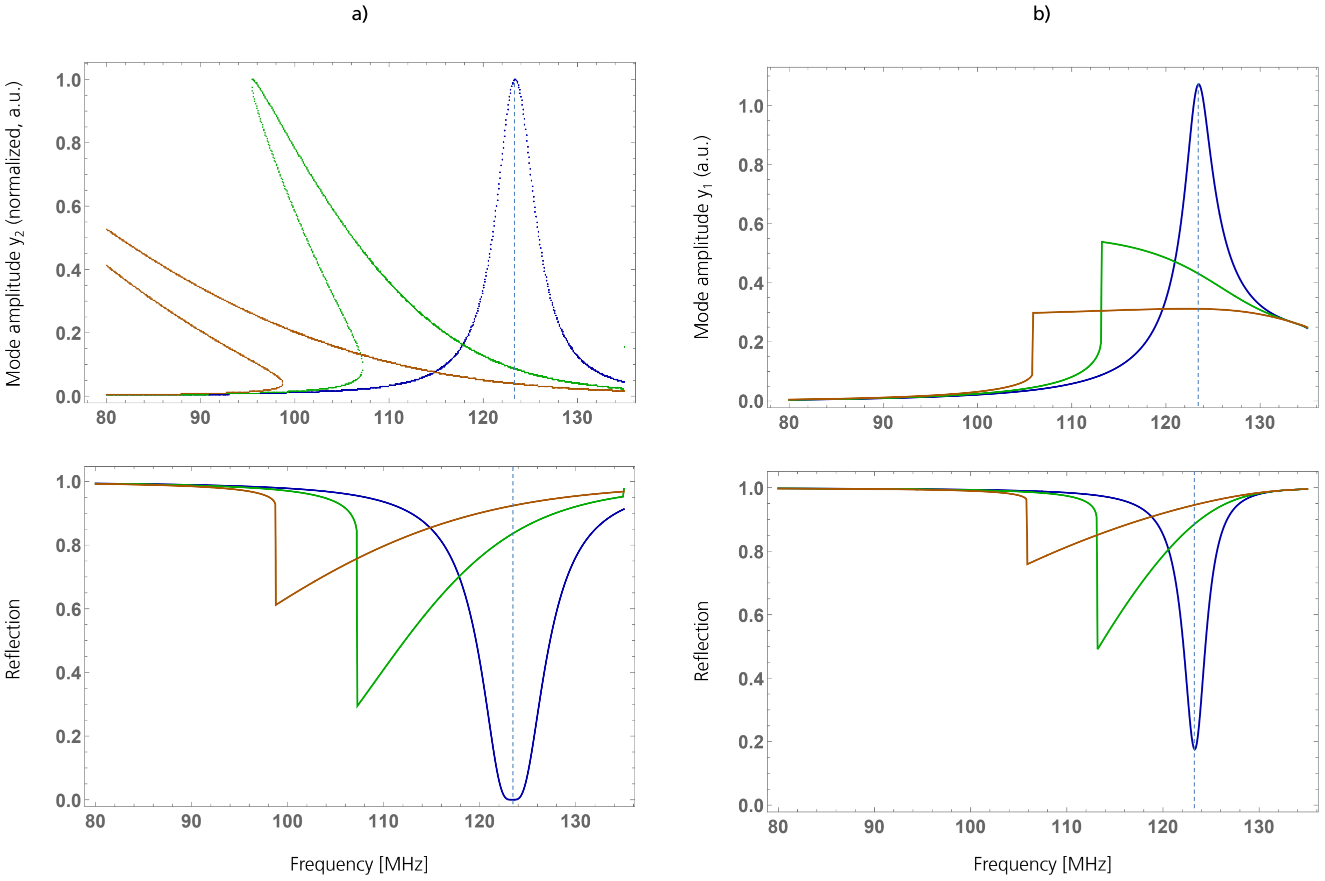

S 1. Theoretical modeling of the non-linear behaviour for different excitation strengths. a) The mode amplitude y_2 and the reflection R of the non-linear resonator are shown for three different incident power levels (isolated tuning resonator with $\Omega_2 = 123.5\,\text{MHz}$, without coupling, i.e., $k = 0$). b) Mode amplitude y_1 and reflection R of the coupled, non-linear metasurface system; compare Fig. 1e in the main text, which shows the S11 measurements with an untuned sniffer coil. For low incident power (blue curves), the structure is resonant at the MR scanner's resonance frequency. At higher incident power (green, brown) the system becomes progressively detuned.

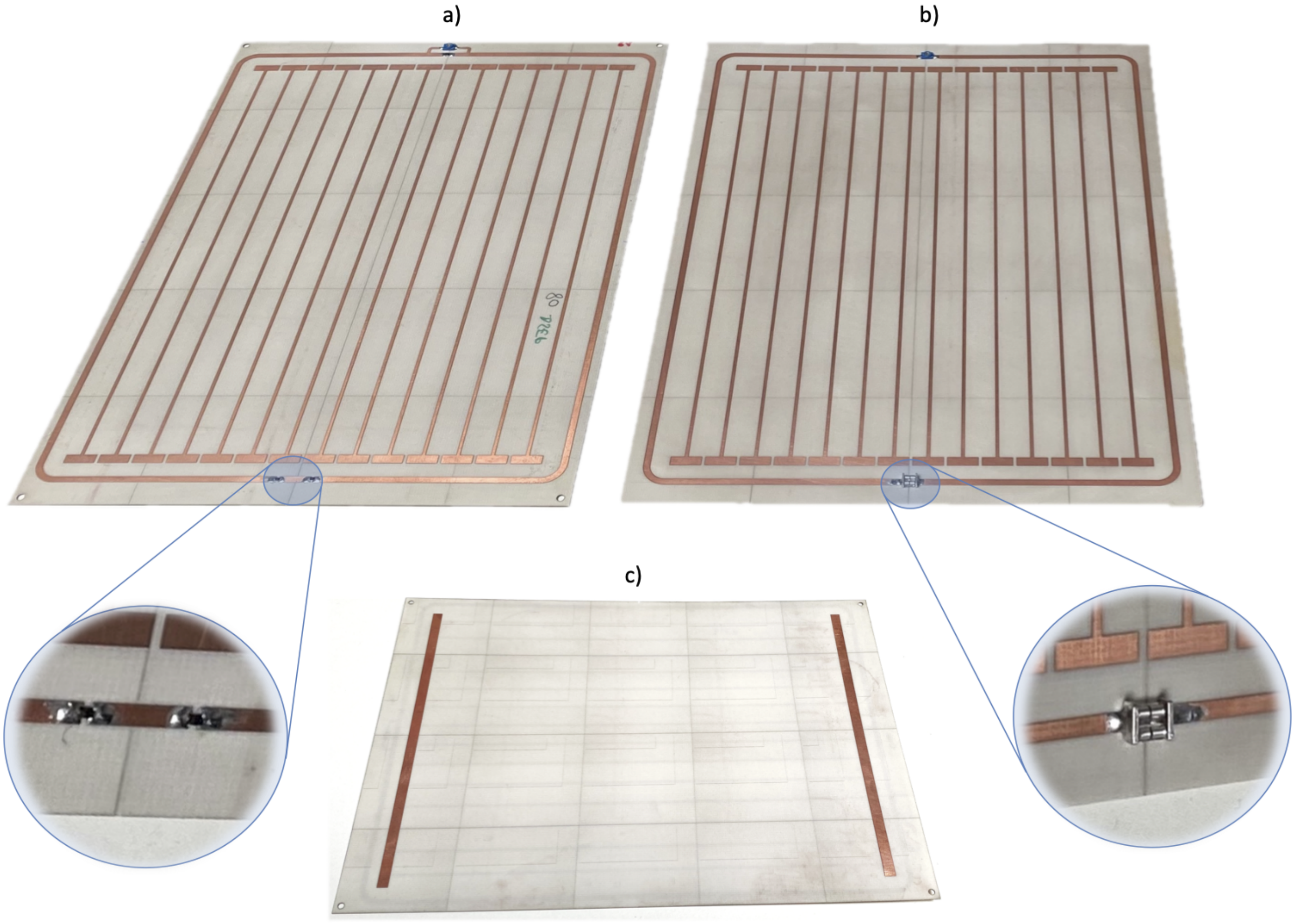

S 2. The two manufactured smart metasurface enhancement plates with a) varactor-loaded tuning resonator and b) limiter-diode-loaded tuning resonator. c) The back of the plate with the ground patches for capacitively coupled wire resonator unit cells is shown, which is the same for both EPs. The ground stripes on the back in combination with the rectangular patches at the end of the wire-resonators on the front form parallel plate capacitors and couple the individual unit cells.

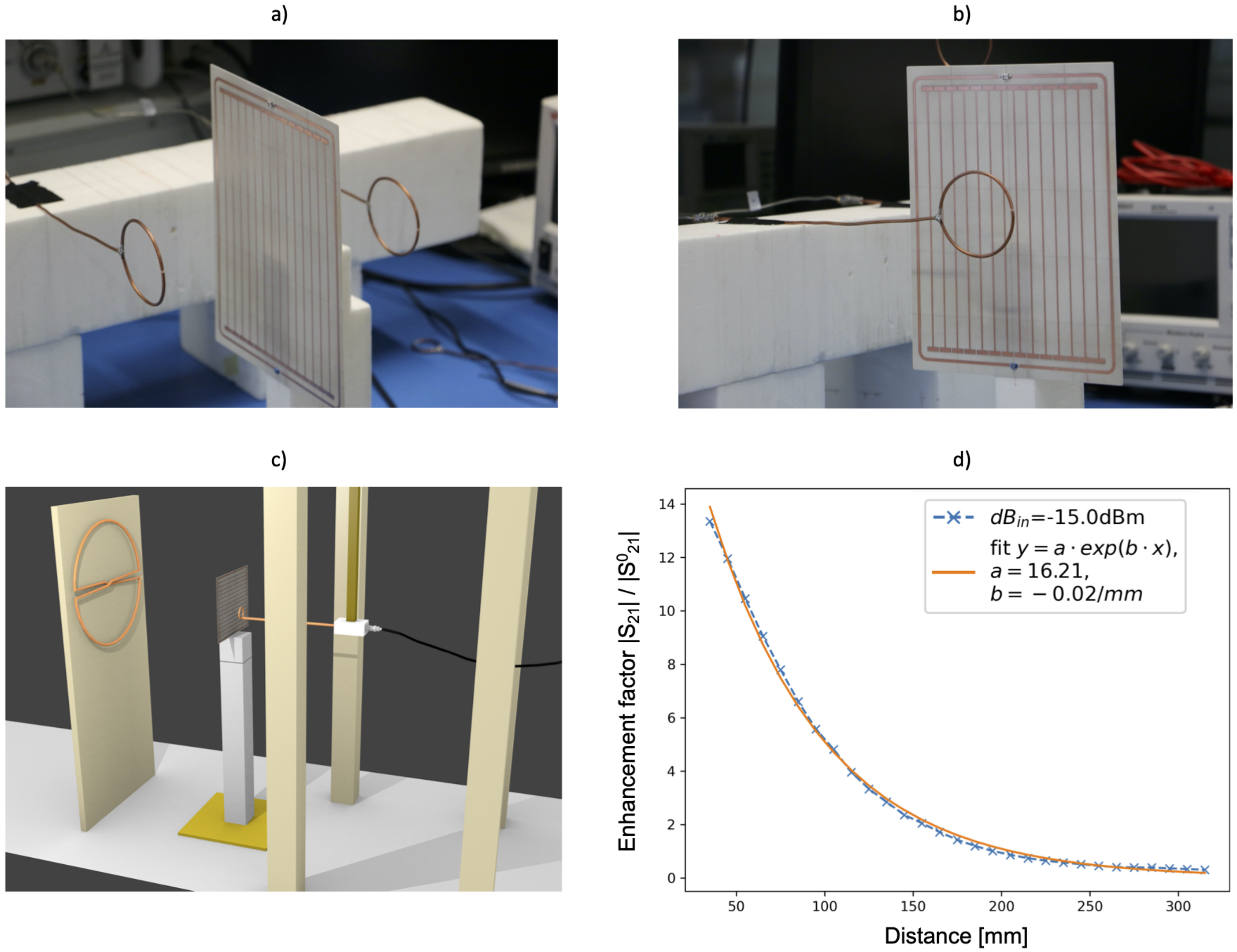

S 3. On-bench characterization of the manufactured prototypes using un-tuned sniffer coils and a vector network analyzer. a) Two sniffer coils positioned for $S21$ measurements. b) A single sniffer coil closer to the EP for $S11$ measurements. c) Schematic of the setup used for measurements of the spatial dependence of scattering parameters. d) Fit of the exponential decay of the enhancement factor as a function of the distance. At $-15\,$dBm input power, we observe an exponential decay of about 0.02/mm.

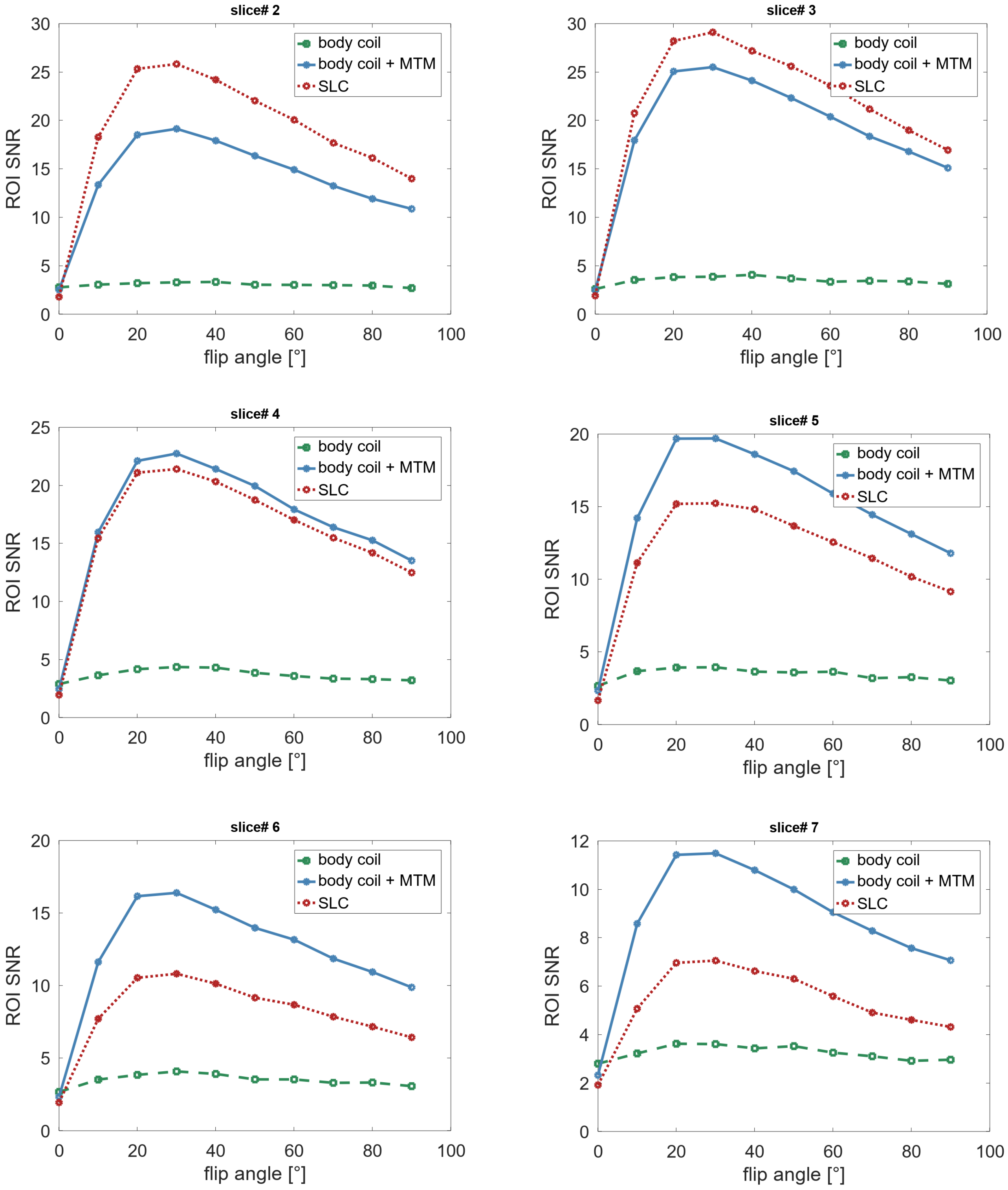

S 4. Additional MRI results for structural images with the Kiwi fruit with $TR = 100\,\mathrm{ms}$. The plots show the SNR in the ROI in different slices (see Fig. 4). The slices are parallel to the metasurface, i.e., the slice number is a measure of distance. The maximum's position is unchanged by the presence of the metasurface in all slices, respectively. This indicates that the EP only influences the Rx field.

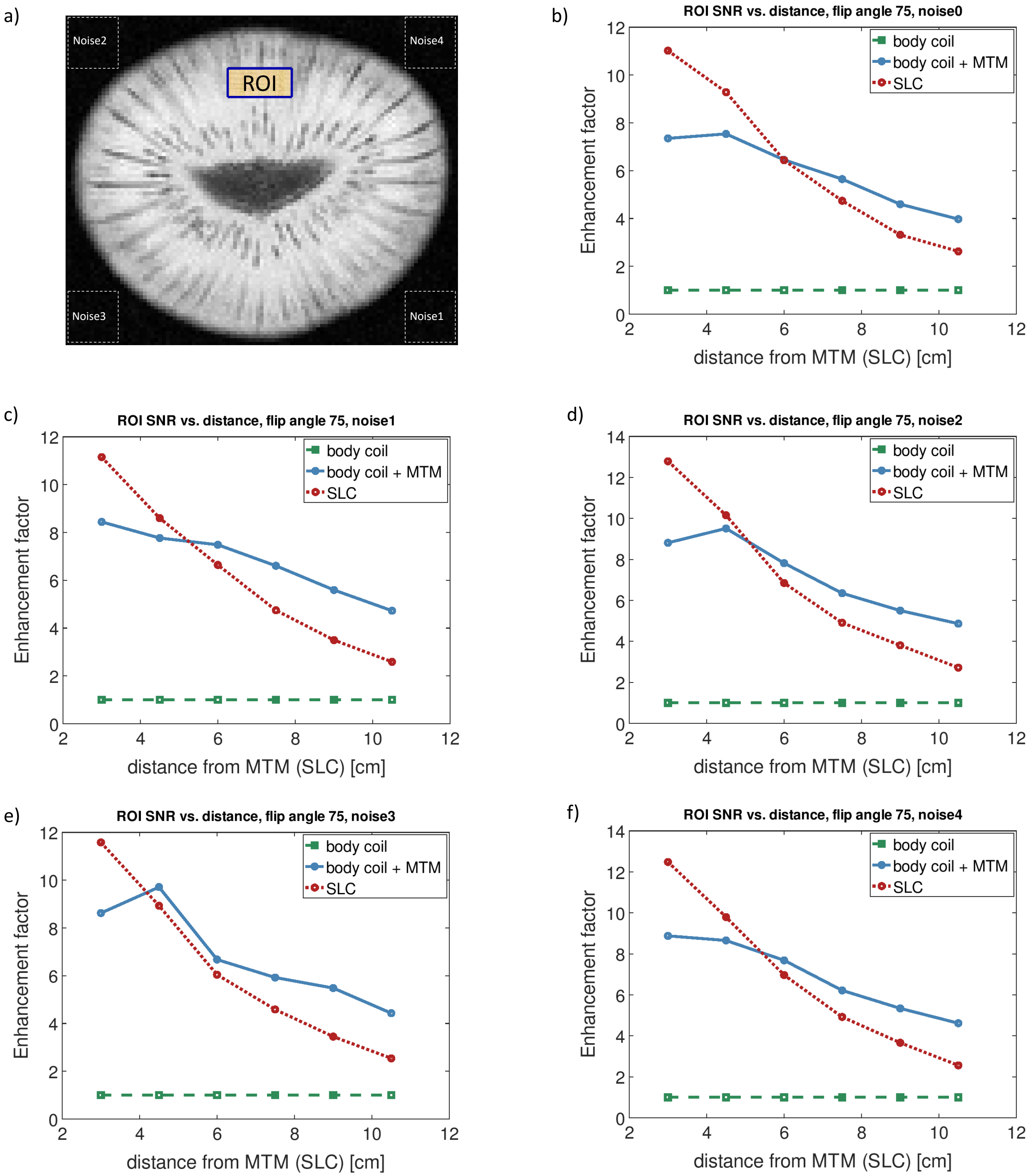

S 5. Additional MRI results for structural images with the Kiwi fruit with $TR = 1\,\mathrm{s}$. The plots show the normalized (w.r.t. the body coil) SNR in the ROI vs. distance from the metasurface for different noise definitions. For noise0, the noise is calculated from the 0 deg flip angle measurements, see the methods section. In all other cases, the noise is the standard deviation in the indicated apparently 'signal-free' areas, respectively. The smart metasurface enhancement factor drops off slower with increasing distance as compared to the SLC. As can be seen, it depends, of course, on the definition of "noise" but the noise as calculated from the 0 deg flip angle scans gives the most conservative measure.

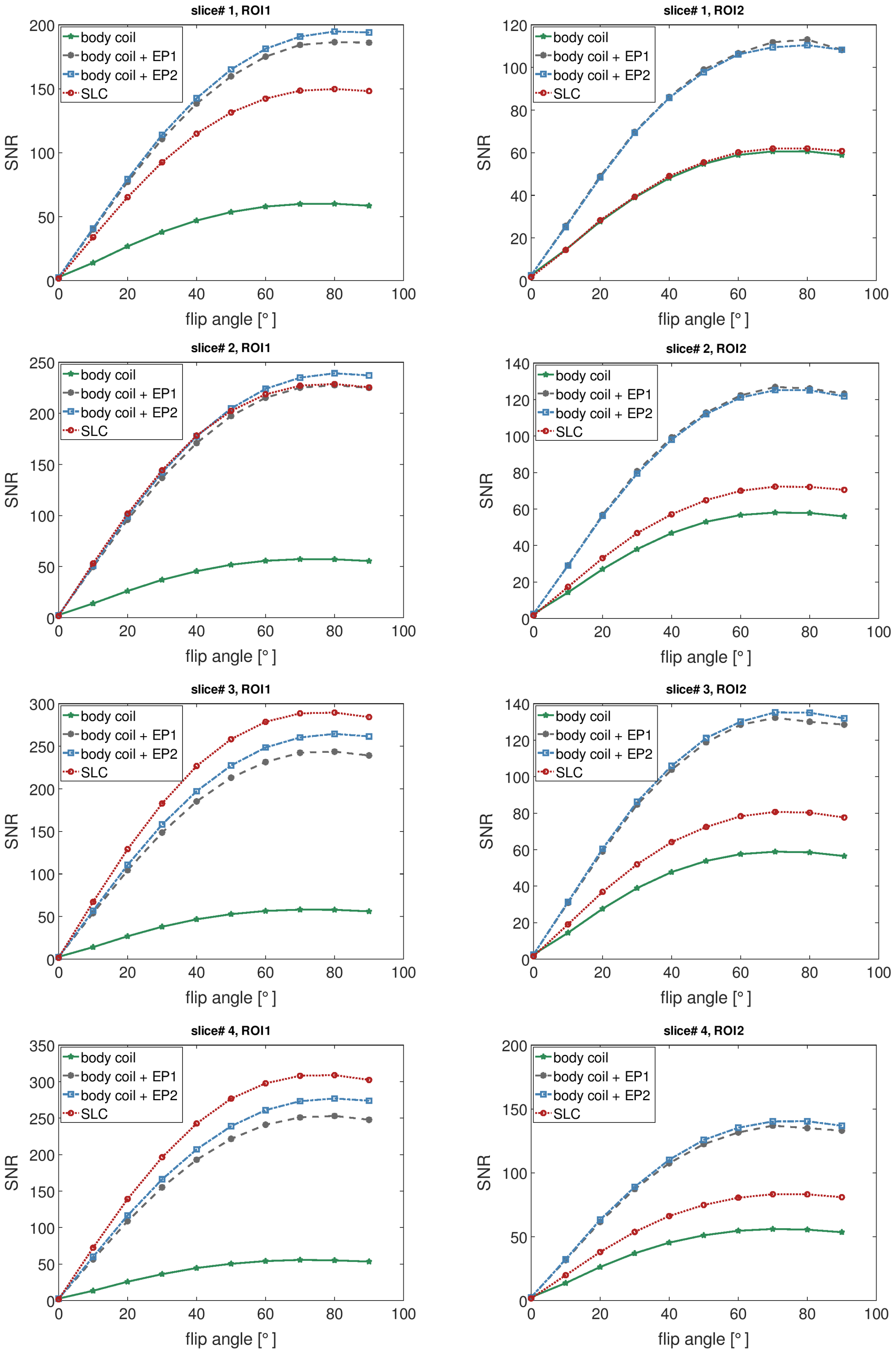

S 6. Additional MRI data for phantom measurements. The SNR is shown in the two ROIs as a function of the flip angle for slices# 1–4. The maximum's position (Ernst angle) is unchanged in presence of either EP, thus, the smart metasurfaces do not influence the Tx field and the SNR increase is purely due to Rx effects.

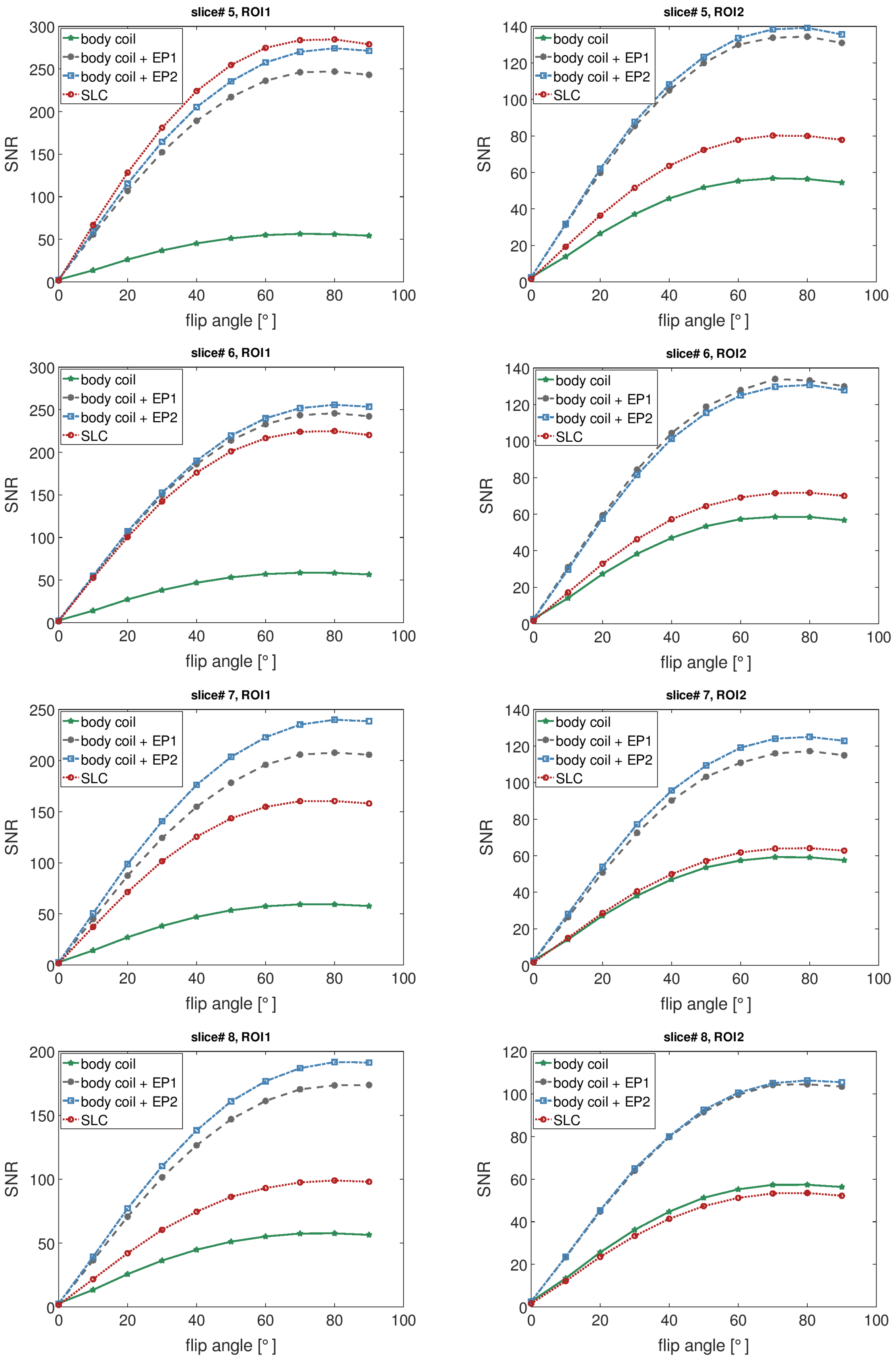

S 7. Extension of the previous figure for slices# 5–8.
